# Nanohydroxyapatite Reinforced Chitosan Composite Hydrogel with Tunable Mechanical and Biological Properties for Cartilage Regeneration

**DOI:** 10.1038/s41598-019-52042-7

**Published:** 2019-11-04

**Authors:** B. Y. Santosh Kumar, Arun M. Isloor, G. C. Mohan Kumar, Inamuddin, Abdullah M. Asiri

**Affiliations:** 1 Polymer Composites Laboratory, Department of Mechanical Engineering, National Institute of Technology Karnataka, Surathkal, Mangalore 575 025 India; 2 Membrane Technology Laboratory, Department of Chemistry, National Institute of Technology Karnataka, Surathkal, Mangalore 575 025 India; 3 Chemistry Department, Faculty of Science, King Abdulaziz University, Jeddah, 21589 Saudi Arabia; 4 Centre of Excellence for Advanced Materials Research, King Abdulaziz University, Jeddah, 21589 Saudi Arabia; 5 Advanced Functional Materials Laboratory, Department of Applied Chemistry, Faculty of Engineering and Technology, Aligarh Muslim University, Aligarh, 202 002 India

**Keywords:** Biomedical materials, Biomedical materials

## Abstract

With the continuous quest of developing hydrogel for cartilage regeneration with superior mechanobiological properties are still becoming a challenge. Chitosan (CS) hydrogels are the promising implant materials due to an analogous character of the soft tissue; however, their low mechanical strength and durability together with its lack of integrity with surrounding tissues hinder the load-bearing application. This can be solved by developing a composite chitosan hydrogel reinforced with Hydroxyapatite Nanorods (HANr). The objective of this work is to develop and characterize (physically, chemically, mechanically and biologically) the composite hydrogels loaded with different concentration of hydroxyapatite nanorod. The concentration of hydroxyapatite in the composite hydrogel was optimized and it was found that, reinforcement modifies the hydrogel network by promoting the secondary crosslinking. The compression strength could reach 1.62 ± 0.02 MPa with a significant deformation of 32% and exhibits time-dependent, rapid self-recoverable and fatigue resistant behavior based on the cyclic loading-unloading compression test. The storage modulus value can reach nearly 10 kPa which is needed for the proposed application. Besides, composite hydrogels show an excellent antimicrobial activity against *Escherichia coli, Staphylococcus aureus* bacteria’s and *Candida albicans* fungi and their cytocompatibility towards L929 mouse fibroblasts provide a potential pathway to developing a composite hydrogel for cartilage regeneration.

## Introduction

Articular cartilage (AC) is a specialized connective tissue of diarthrodial joints, which provides smooth, lubricating surface for articulation and to ease the transmission of the load with a low frictional coefficient. Cartilage lesions due to aging, traumatic injury have always made significant problems due to its limited potency for inherent self-healing, which means the functional cartilage is hard to reconstruct *in situ*^1^. Microfracture, Osteochondral Autograft, Autologous Chondrocyte Implantation (ACI) and Allograft Transplantation are few clinical technics for cartilage restoration^2^. However, the donor deficiency and disease transfer are the common problems and few authors have reported that, these clinical surgeries are limited to lesion thickness was less than 3 mm^3^. Knee arthroplasty is a prominent treatment in practice. However, still it is not free from many drawbacks; first, it needs to amputate a lot of healthy bone and once if surgery fails, the next surgery is very hard to conduct. Second, elastic mismatch between the implant and patient bone causes aseptic loosening. Third, due to the rubbing of polymer over metal, fibers may leach out which leads to chronic infections^4–6^. Therefore, there is an impetus for developing cartilage substitutes to reconstruct the tissue function.

Hydrogel, a three-dimensional cross-linked hydrophilic polymer network owing to the similarity in the structure and some attributes of soft tissue is widely used to develop scaffolds for tissue engineering^7,8^. Synthetic polymeric hydrogels such as polyvinyl alcohol, Polyethylene glycol, and Polyacrylamide are commonly used synthetic for hydrogels preparation^9–11^. However, the biocompatibility and cell proliferation ability severely restrict their application *in vivo*. Protein-based natural polymers are advantageous, because they contain ligands that can be confessed by cell-surface receptors^12–14^. Among them, Chitosan (CS) has received much attention owing to its excellent biocompatibility, biodegradability, nontoxicity and other chemical properties due to the amino and hydroxyl functional group^15–18^. However, the low mechanical strength caused by higher hydrophilicity hampers their structural stability *in vivo*. The metal and metal oxide nanoparticles such as silver^19^, titanium dioxide^20^, silica^21^ are few reinforcements to strengthen the CS. Still, there are some shortcomings such as these nanocomposite lowers alkaline phosphate activity, osteopontin secretion which suppresses the mineralized tissue formation^22^. Overdose of titanium can cause carcinogenic problems^23^ and the uneven sized nanoparticulate reinforcements can cause increased friction on the bone-cartilage interfaces^24^.

As the major component of inorganic bone tissue is hydroxyapatite (HAp), might be a promising reinforcement for biomedical hydrogels. However, most of the HAp used currently as coatings and orthopedic implants, and decidedly less information is available on the influence of HAp on interfacial bonding of soft and hard tissues. The properties such as particle size, microstructure, morphology and dimensional anisotropy are the critical parameters for optimization and application. One way to improve the mechanobiological characteristics of HAp is developing one-dimensional Hydroxyapatite Nanorod (HANr). This nanorod morphology not only favors the adhesion and proliferation of osteoblast but also increase alkaline phosphatase activity of marrow stromal cells^25^. Further, the rod-shaped HAp morphology had higher polar moment of inertia, which accords structural stability for nanocomposite hydrogel^26^. Besides, the human bone is consisting of parallel rod-like hydroxyapatite structures with a cross-section of 30–60 nm diameter and 100–1000 nm in length counteract mechanical strength and flexibility^27^.

As per our observations, till now there are no reports on HANr reinforced chitosan nanocomposite hydrogel for tissue-engineered articular cartilages. In view of the same, it was planned to prepare the hydroxyapatite nanorod from cuttlefish bone through the mechanochemical method and chitosan was blended with HANr to match the requirements. This CS/HANr composite network system provides state-of-the-art, where a part of the polymeric network tries to improve biocompatibility and another part anchors mechanical strength. This opens a new window to tailor the property of interest. To bind the composite structure, Glutaraldehyde (GA) was used as an active chemical cross-linker, which forms an intra-interchain covalent bonding. However, it has moderately cytotoxic that might spoil osteoconductivity. But, it is believed that all the functional groups are actively participating in crosslinking, or they blocked by the molecules of amino acids and proteins of living organism serum^28^.

### Materials

Low molecular weight Chitosan (CS, the degree of deacetylation ≥75%) was purchased from Sigma-Aldrich, India. The cuttlefish bone (CB) was procured from Blue Water Foods and Exports Pvt. Ltd. Mangalore, India. Analytical grade phosphoric acid (H_3_PO_4_) was purchased from Merck, India. Glutaraldehyde (~25% aqueous solution) was procured from Spectrochem, India. Mammalian L929 normal mouse fibroblast cell lines were supplied by National Centre for Cell Science (NCCS) Pune, India. Dulbecco’s Modified Eagle’s Medium (DMEM), Fetal Bovine Serum (FBS), Phytohaemagglutinin (PHA) and MTT reagents were procured from Himedia Laboratories Mumbai, India. Cell freezer Dimethyl sulfoxide (DMSO) was supplied by Sigma Aldrich, India. All other chemicals and solvents were of analytical or pharmaceutical grade and used as received.

### Hydroxyapatite nanorod preparation

Cuttlefish bones (CB) were washed with the boiling water to remove the fleshy material and then dried in an incubator at 70 °C for 24 h. The sterile bones were powdered and sieved in a sieve shaker. 2 g of CB powder was dissolved in 100 mL of demineralized water under intense magnetic stirring (10 min, 450 rpm) at 70 °C followed by the addition of 550 µL orthophosphoric acid (H_3_PO_4_) in order to maintain a stoichiometric molar ratio of 1.67 (Ca/P). Stirring was continued for another 10 h and the slurry thus obtained was dried and calcined in a muffle furnace at 800 °C with a heating rate of 5 °C/min for 4 h.

### Preparation of CS/HANr composite hydrogel

The CS/HANr composite hydrogel was prepared by dissolving 1 g of CS in 0.1 M aqueous acetic acid solution. HANr with different concentration (0.5, 1.0, 1.5, 2.0, and 2.5 wt% named as 0.5HANr, 1HANr, 1.5HANr, 2HANr and 2.5HANr) were suspended in 50 mL demineralized water for 1 h in a probe sonicator. The suspension was then slowly added to the CS solution and is diluted with demineralized water to a final volume of 100 mL under intense magnetic stirring (10 h, 400 rpm). Then, glutaraldehyde (0.5% v/v) was added in a dropwise manner with constant stirring and the solution mixture was poured into silicon mould at −15 °C for 8 h. Then, samples were washed several times with 70% ethanol until neutral pH was obtained. Finally, samples were washed with sterile water and stored at 20 °C in the demineralized for further characterization^29^.

### Morphological and IR analysis of the composite hydrogel

To analyze the pore size, distribution and morphology, SEM (JSM-6380LA, JOEL, Japan) micrographs were obtained at an operating voltage of 5 kV. The average pore size was determined by the JAVA based image processing program ImageJ for scaffold imaging. Swollen gels were lyophilized and cryofractured in the liquid nitrogen. These steps are crucial for obtaining a porous gel network. Prior to the observation, hydrogels were sputter-coated with a gold layer of about 100 A° to avoid charging effect. The structural modification of composite hydrogel was identified by attenuated total reflectance fourier transform infrared (ATR-FTIR) spectroscopy from the Bruker alpha instrument at an operating wavelength range of 4000–500 cm^−1^ with a resolution of 8 cm^−1^.

### Swelling studies

For welling study, the lyophilized hydrogels (W_d_) were incubated in distilled water at 37 °C. Then samples were taken out and weighed (W_s_) after soft surface wiping with absorbent paper at regular interval of time until equilibrium swelling was reached. The Equilibrium Swelling Ratio (SR) is defined as the ratio of swollen weight to the initial weight. To minimize the experimental error, all the experiments were performed in triplicate and their average value was recorded^30^.

$${\rm{SR}}=\frac{({{\rm{W}}}_{{\rm{s}}}-{{\rm{W}}}_{{\rm{d}}})}{{{\rm{W}}}_{{\rm{d}}}}\times {\rm{100}}$$

### Contact angle measurement

The hydrophilicity of the composite samples was measured by assessing the angle formed between a drop of fluid (Phosphate Buffer Saline, PBS (pH 7.2)) and the hydrogel surface. The contact angle was determined using water contact analyzer (Kruss, Germany). Composite hydrogels of 0.5 mm thick layer were cast on the glass substrate to get the relatively flat hydrogel surface. For minimizing the experimental error, three readings were taken for each sample and their average value was reported^31^.

### Compression and cyclic compression test

The unconfined compression and cyclic loading-unloading compression tests were performed in the universal testing machine (Mecmesin MultiTest 10-i micro UTM, London, UK) with 1000 N load cell. The cylindrical samples of 10 mm diameter and 7 mm height were used with a crosshead speed of 5 mm/min for unconfined compression test and 20 mm/min cyclic loading-unloading compression tests. Prior to the test, a preload of 0.01 N was applied to confirm the clear contact between the compression plates and the hydrogel. The force and deformation data were used and Stress vs. Strain graph was plotted. The Elastic modulus (E), Stress-at-break (σ_max_) and Strain-at-break (ε_max_) were determined from the curves^32,33^.

### Rheological test

The rheological behavior of composite samples was performed at 37 °C in an MCR 302 Anton Paar Rheometer. An oscillatory frequency sweep test was carried out in the frequency range of 0.1–100 Hz with 1% constant strain. The components of complex modulus such as storage modulus (G’) and loss modulus (G”) were investigated^29^.

### Microbial study

The microbes used in this study including standard strains of *Escherichia coli (E. coli, ATCC 25922), Staphylococcus aureus (S. aureus, ATCC 25923)* and *Candida albicans (C. albicans CCRC21538)*. Nutrient agar and Nutrient Broth was procured from Sisco Research Laboratories, India and HiMedia Laboratories, India respectively. All the strains were incubated at 37 °C for 12 h.

### Growth inhibition test

The antimicrobial activity was evaluated according to the growth inhibition assay using the modified method explained elsewhere^34^. In brief, 150 µL of nutrient broth and 50 µL of diluted bacterial or fungal culture were taken in a 96 well culture plate. The initial absorbance at 600 nm was measured in a Thermo scientific Multiscan® plate reader and considered as initial bacterial/fungal concentration. Then, 10 mg of triplicate CS/1.5HANr hydrogel samples were added to the respective well and incubated it overnight at 37 °C. The inoculum was then monitored for changes in the absorbance at 600 nm (OD_600_). The well without any sample was considered as control. The percentage growth compared to the control was calculated for each of the sample.

### Measurement of cytotoxicity

The cytocompatibility of the hydrogel was assessed by determining the viability of the L929 mouse fibroblast cells in response to the conditioned media using MTT assay. Briefly, hydrogel discs (10 mm diameter, 2 mm thick) were sterilized in 70% ethanol followed by washing in a sterile PBS. Fibroblast L929 cells were seeded on the hydrogel surface (50,000 cells/well) in a 24-well plate with DMEM supplemented with 10% FBS and incubated at 37 °C for 72 h in 5% CO_2_. Then, media was replaced with MTT to a final concentration of 5 mg/mL and incubate for another 3 h. Finally, 100 µL of DMSO was added with gentle stirring in a gyratory shaker. The medium without any treatment was considered as control (100%) and cell activator Phytohaemagglutinin (PHA) treated hydrogel was considered as standard^35,36^. The optical density (OD) of the media was measured at 570 nm to determine the cell viability, according to the equation:

$${\rm{Cell}}\,{\rm{viability}}\,( \% )=\frac{{{\rm{OD}}}_{{\rm{test}}}-{{\rm{OD}}}_{{\rm{blank}}}}{{{\rm{OD}}}_{{\rm{control}}}-{{\rm{OD}}}_{{\rm{blank}}}}$$

where, OD_test_, OD_control_ and OD_blank_ are the optical densities of cells incubated with hydrogel, DMEM with cells and DMEM without cells respectively.

### Statistics

All quantitative results were obtained from triplicate samples and data were shown as a mean ± standard deviation. Statistical significance is calculated using the one-way ANOVA test. A value of p < 0.05 were considered as statistically significant.

## Results and Discussion

### Characterization of hydroxyapatite nanorod

**Figure 1:**
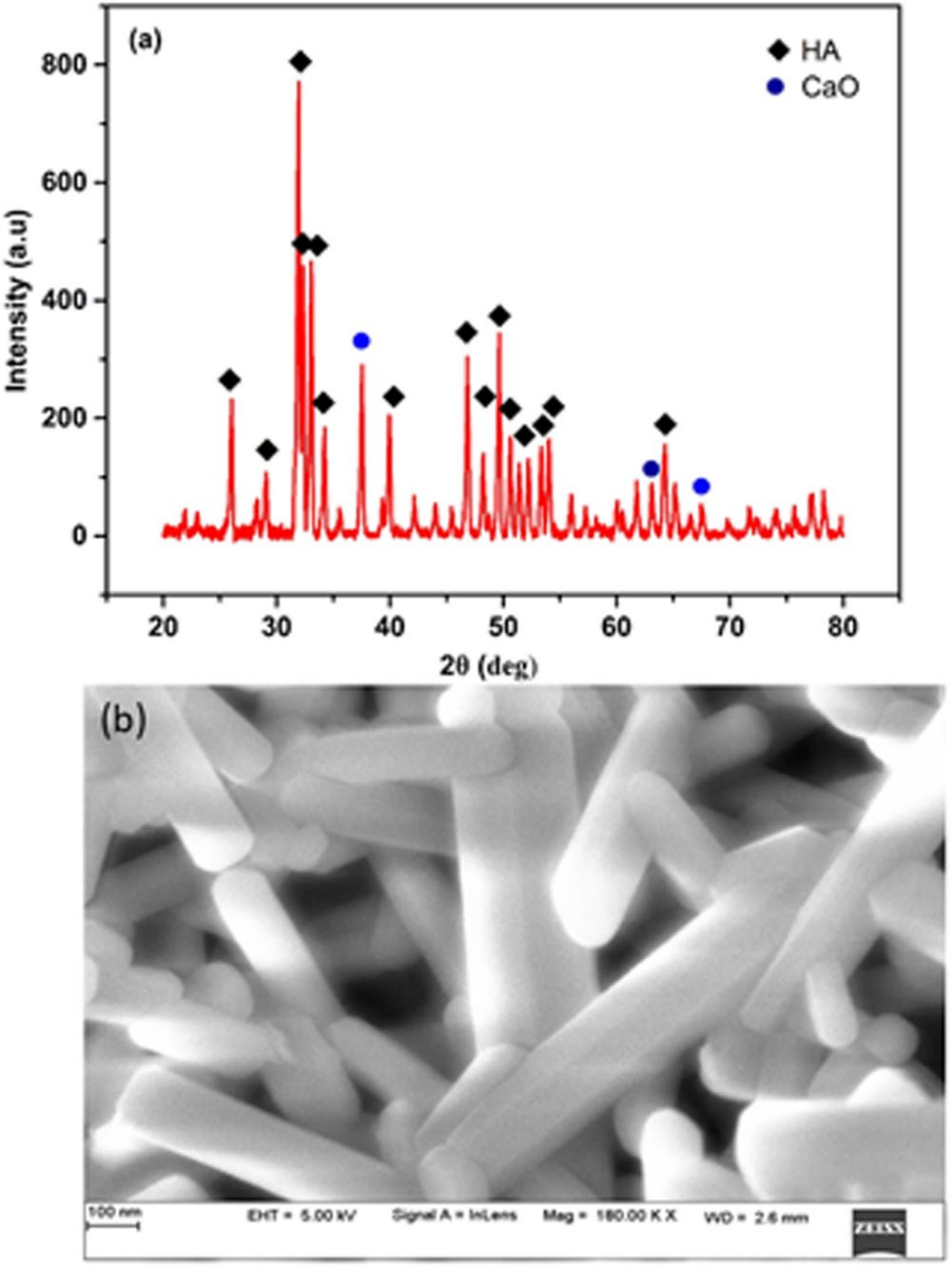
(**a**) X-ray diffraction spectra and (**b**) SEM of synthesized hydroxyapatite nanorod.

The XRD spectrum of hydroxyapatite has been presented (Fig. 1a). The most common form of calcium phosphate is hydroxyapatite which crystallizes in the hexagonal system. The principal hydroxyapatite phase was confirmed with JCPDS card no: 09–432^37^. The presence of well-resolved major intensity peaks in the range 2θ = 20–35° confirms the good crystallinity of hydroxyapatite. Besides, there could be a certain amount of retained calcium oxide confirmed with JCPDS card no: 37–1497^38^. The morphology of the synthesized HANr was analyzed from the results of SEM (Fig. 1b). The patterns showed the rod-shaped morphology existed as numerous arrangements of uniform bundles of one-dimensional nano-rod like structure with an average size of 186 ± 12 nm diameter and 1000–1500 nm of length.

### Macroscopic appearance

**Figure 2:**
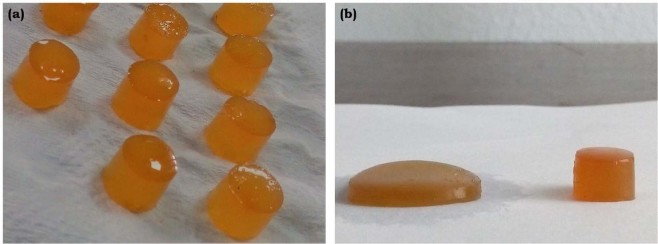
Photographs of (**a**) CS and (**b**) CS/HANr composite hydrogel.

The photograph of the developed hydrogel is as shown (Fig. 2). It has been observed that a pure CS hydrogel is yellowish, glowing and semi-transparent. Whereas, the addition of the HANr relatively turns the gel into pale yellow and opaque.

### Morphology and IR analysis

**Figure 3:**
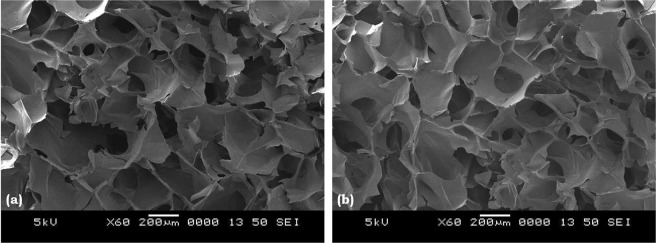
SEM images of (**a**) CS and (**b**) CS/1.5HANr composite hydrogel.

The internal morphology of the transverse sectioned lyophilized hydrogels is as shown (Fig. 3). All the hydrogels showed the porous microstructure with interconnected porosity and the average pore size of CS and CS/1.5HANr are 244 µm and 181 µm respectively. This interconnected porosity is essential for cartilage scaffolds to diffuse nutrients and excretions in the biological environment^39^. These tiny pores do not affect the mechanical properties of load-bearing hydrogels. Despite, they disburse load more evenly and act as a barrier for crack propagation, which increases the fatigue strength. Further, it lowers the permeability and hence more barrier to fluid flow and thus increasing the fluid load support^40^. In brief, CS/1.5HANr composite hydrogels are having combined micro and nano porosity which makes hydrogel better for repair and regeneration of cartilage tissue^41^.

**Figure 4:**
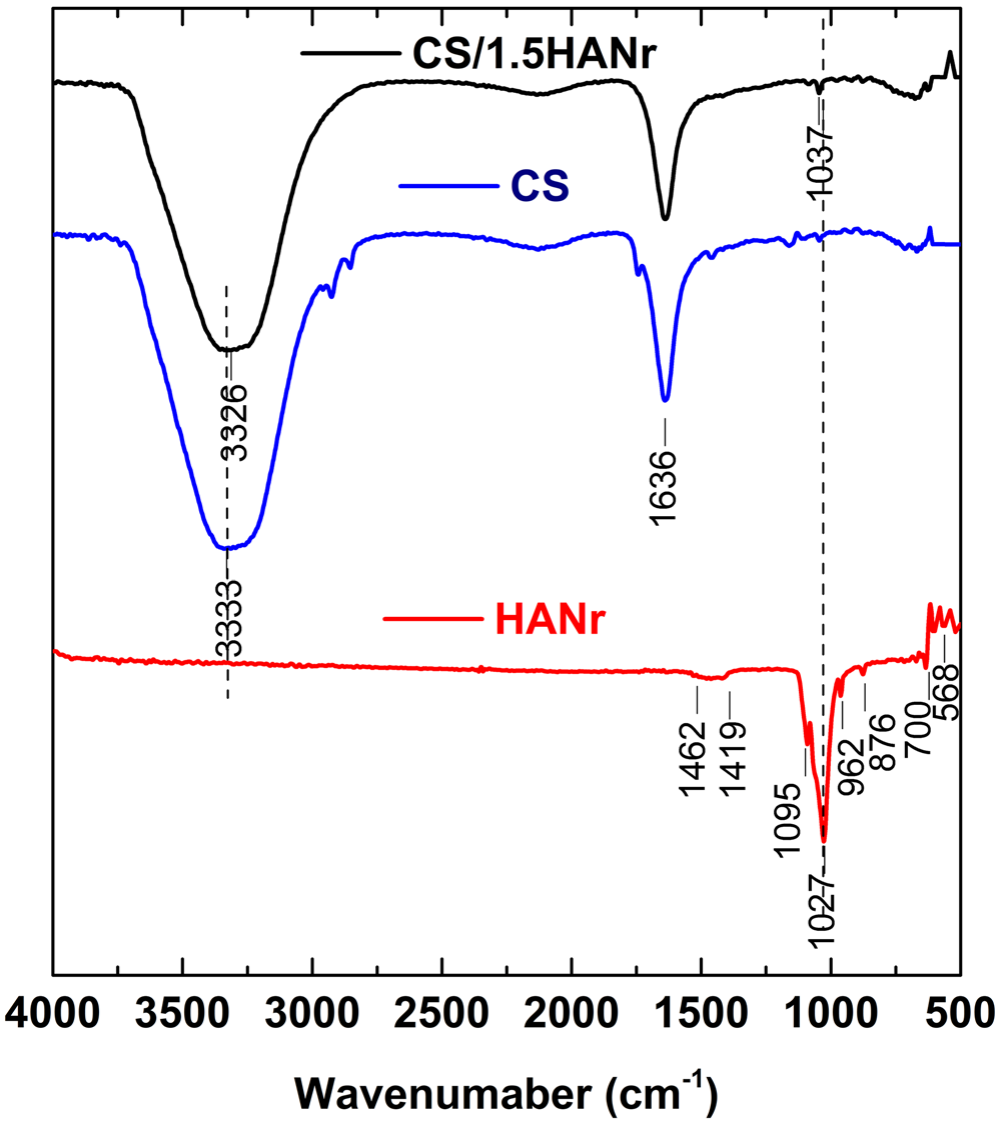
FTIR spectra of (**a**) HANr (**b**) CS and (**c**) CS/1.5HANr hydrogel.

The structural modification of the composite hydrogel due to hydroxyapatite reinforcement was identified by FTIR spectrum (Fig. 4). The characteristic bands of HANr were located at 568 cm^−1^, 700 cm^−1^, 876 cm^−1^ and 962 cm^−1^, 1027 cm^−1^, 1095 cm^−1^, 1419 cm^−1^ and 1462 cm^−1^ ^42,43^. Similarly, chitosan peaks at 1636 cm^−1^ region are owing to the C=N bond of Schiff base due to the interaction of free amine groups of chitosan and aldehyde functionality of glutaraldehyde and peak at 3333 cm^−1^ is attributed to the O-H and N-H symmetrical vibration^44^. For composites, a slight shift of the asymmetric stretched phosphate band from 1027 cm^−1^ to 1037 cm^−1^ was observed. Additionally, a characteristic CS peak due to OH stretch was shifted to 3326 cm^−1^. The shift of IR adsorption bands towards lower wavenumber in CS/1.5 HANr composite hydrogel in comparison to CS hydrogel suggests the formation of hydrogen bonding between CS and HANr^45^.

**Figure 5:**
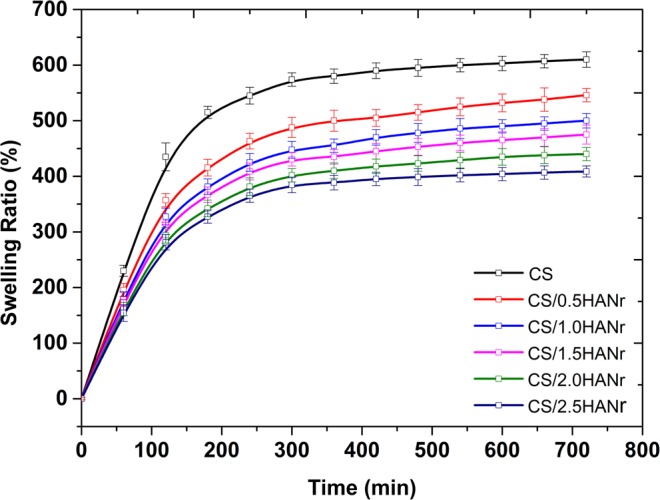
Swelling kinetics of CS and CS/HANr composite hydrogel.

Fluid absorption or swelling are the preliminary requirements of tissue engineering implants. For hydrogels, swelling and amount of water content are mainly related to the amorphous region and free hydroxyl (OH) groups in the polymer. The equilibrium swelling potency of chitosan and its composite hydrogels are summarized in Table 1. The introduction of hydroxyapatite could change swelling characteristics (Fig. 5). As expected, the swelling ability of hydrogel decreases with increasing hydroxyapatite concentration. This could be due to the reinforcement that makes an additional hydrogen bonds as confirmed with FTIR spectra, which arrest the free movement of the polymer chain. Hence more the reinforcement, lesser is the space to hold the water. The surface wettability of biomaterials influences the cell adhesion, proliferation and differentiation on the surface of the scaffold. The maximal cell adhesion is for moderate hydrophilicity and the optimum hydrophilicity for culturing of fibroblasts is estimated to be 55–70° ^46^. The contact angle of all the hydrogels are summarized in Table 1 are lying within this range which assist the fibroblast growth.

**Table 1:** Typical physical properties of CS/HANr composite hydrogel.

Sample	Equilibrium Swelling Ratio (SR, %)	Contact angle°
CS	612 ± 18	66.5 ± 0.3
CS/0.5HANr	546 ± 12	64.1 ± 0.2
CS/1HANr	500 ± 13	63.6 ± 0.3
CS/1.5HANr	475 ± 17	63.2 ± 0.1
CS/2HANr	440 ± 11	61.8 ± 0.4
CS/2.5HANr	409 ± 14	60.7 ± 0.2

### Compression, cyclic compression and fatigue test

**Figure 6:**
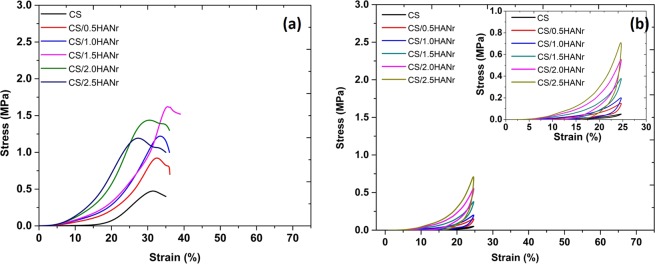
(**a**) Unconfined compression stress-strain curve of composite hydrogel and (**b**) Typical cyclic loading-unloading compression curve of CS/HANr composite hydrogel.

The unconfined compression stress-strain curves of chitosan and its composite hydrogels are as shown (Fig. 6a). These curves corresponds to the behavior of non-linear and viscoelastic solids^47^. As the chitosan hydrogel is stressed, the entangled polymer chains absorb the load and get reoriented, and the interstitial fluid begins to drain out. During this, a small load is enough for substantial deformation (i.e. there no significant stress up to 15% strain). As loading continues, the reorientation tends to be uniform and friction due to interstitial fluid causes the hardening effect of the polymer, which needs an additional effort to increase the strain. In composite hydrogels, an early raise of compressive stress in comparison with chitosan hydrogel is attributed to the reinforcement, can develops the secondary crosslinking points which tries to arrest the free movement of polymer chains and makes hydrogel stiff. The compressive mechanical properties of the hydrogels are summarized in Table 2. Pure chitosan hydrogel shows the least compression strength of 0.4 ± 0.07 MPa with a significant deformation of 33%. However, for composites, it shows a bimodal trend i.e. the compression strength was increases up to 1.62 ± 0.02 MPa for hydrogel having 1.5 wt% of HANr and as HANr concentration increases above 1.5 wt%, a downturn in the compression strength due to the agglomeration.

**Table 2:** Compression mechanical properties of CS/HANr composite hydrogel.

Composition	Compression strength, σ (MPa)	Compressive Strain, ε (%)	Compression Modulus, E (MPa)
CS	0.40 ± 0.07	33.0 ± 1.12	0.29 ± 0.06
CS/0.5HANr	0.90 ± 0.07	34.1 ± 0.95	0.98 ± 0.02
CS/1.0HANr	1.25 ± 0.03	34.6 ± 1.25	1.28 ± 0.05
CS/1.5HANr	1.62 ± 0.02	35.2 ± 1.49	1.73 ± 0.08
CS/2.0HANr	1.43 ± 0.05	28.5 ± 1.18	1.88 ± 0.01
CS/2.5HANr	1.20 ± 0.03	27.2 ± 2.25	2.03 ± 0.06

To explore the mechanism of the synergistic effect of reinforcement on the viscoelastic characteristics of the hydrogel in the loading condition close to the working condition of joint cartilage, the cyclic loading-unloading compression tests were performed at a crosshead speed of 20 mm/min with a fixed strain of 25% (maximum strain induced in the joint is 20%^48^). The basic facts about these materials are the decompression path of the curve is not following its compression path. It may be above or below depending on the strain rate^49^. Figure 6b compares the cyclic loading-unloading curves of chitosan and its composite hydrogels reveal their energy dissipation capacity. The elastic modulus of the hydrogels is summarized in Table 2. The elastic modulus of pure chitosan hydrogel is 0.29 ± 0.06 MPa and it increases with increasing HANr reinforcement demonstrates the good interfacial compatibility between matrix and the reinforcement. This increasing reinforcement concentration makes hydrogel stiffer and could dissipate energy by showing large hysteresis loop, while there was hardly a loop visible for CS hydrogel^11^.

**Figure 7:**
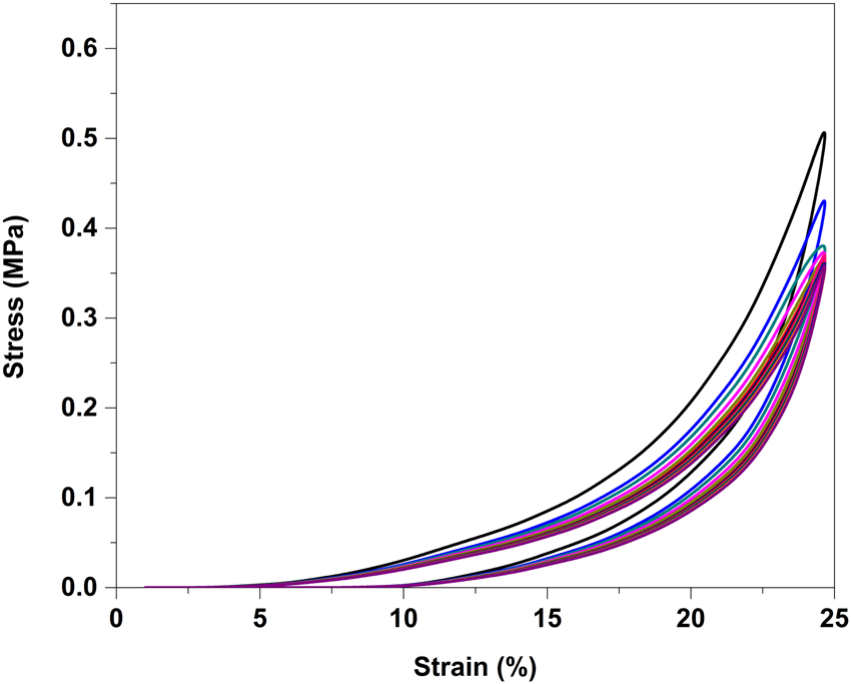
Cyclic loading-unloading curve of CS/1.5HANr composite for hundred cycles.

Further, to study the fatigue strength of the CS/1.5HANr hydrogel, a hundred cycles of loading-unloading compression tests were performed at rate of 20 mm/min with a constant strain of 25% as shown (Fig. 7). As far as one can analyze the cyclic curves presented, in the first compression cycle, a part of the chemical crosslinking had been broken, large hysteresis loop occurred. Due to the permanent collapsing of the chemical crosslinking, for the next four cycles the hysteresis loop size decreasing continuously and then loops were almost completely overlapped for succeeding cycles demonstrating that the composite hydrogel CS/1.5HANr possessed a rapid self-recovery and fatigue resistance^33^.

### Rheological studies

**Figure 8:**
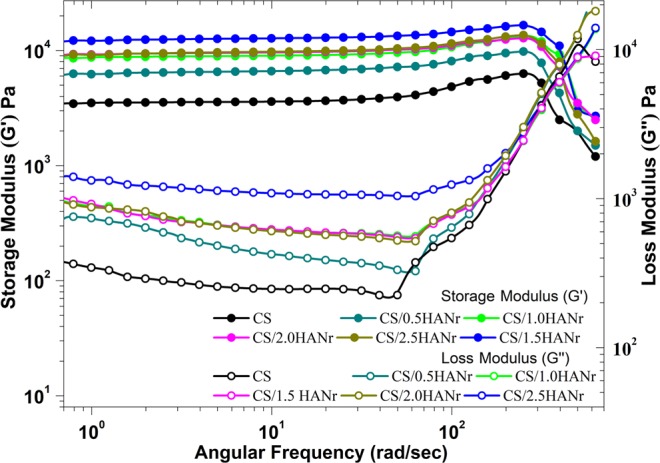
Rheological properties of chitosan and its composite hydrogels as a function of angular frequency.

The variation in viscoelasticity of hydrogels with hydroxyapatite was characterized by rheological experiments. The correlation of storage (G’) and loss modulus (G”) of the composites as a function of angular frequency was depicted (Fig. 8). It mainly depends on the coordinate bonds between the amino groups of CS and aldehyde functionality, electrostatic and hydrogen bonding between matrix and the reinforcement in the network. Initially, the G” values of all composites were less than the G’ values for an angular frequency less than 350 rad/sec signifies all hydrogels are predominantly elastic behavior rather than the fluid-like state^29^. Both, G’ and G” values increases with increasing hydroxyapatite, indicates good interfacial compatibility between chitosan and hydroxyapatite. The storage modulus of hydrogel increases with increasing frequency, which is similar to the changing rule with the frequency of natural cartilage, signifies that physically cross-linked hydrogels have superior mechanical strength and could withstand shear stress caused by synovial fluid in the di-arthrodial joint. As an angular frequency increases above 350 rad/sec, G” values raised above G’ shows that the entanglements developed by generated hydrogen bonding between deprotonated –NH_2_ and –OH groups; and interaction of N-acetyl and main polysaccharide backbone of CS were broken and signified the end of the viscoelastic region at the critical stress^50^.

### Antimicrobial properties of the CS/HANr hydrogel

**Figure 9:**
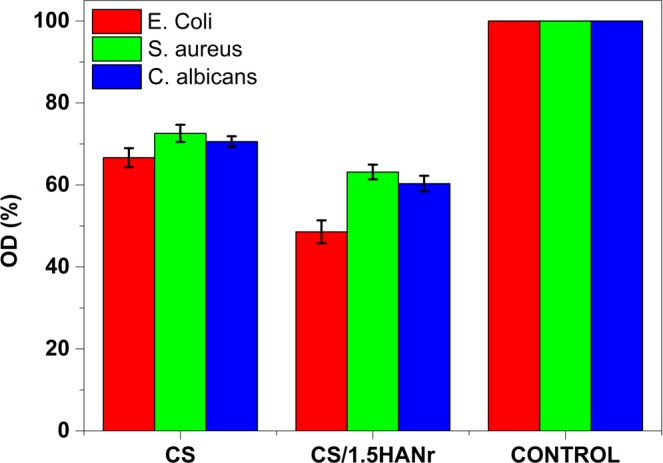
Antimicrobial activity of CS and CS/1.5HANr composites against *Escherichia coli*, *Staphylococcus aureus* and *Candida albicans*.

**Figure 10:**
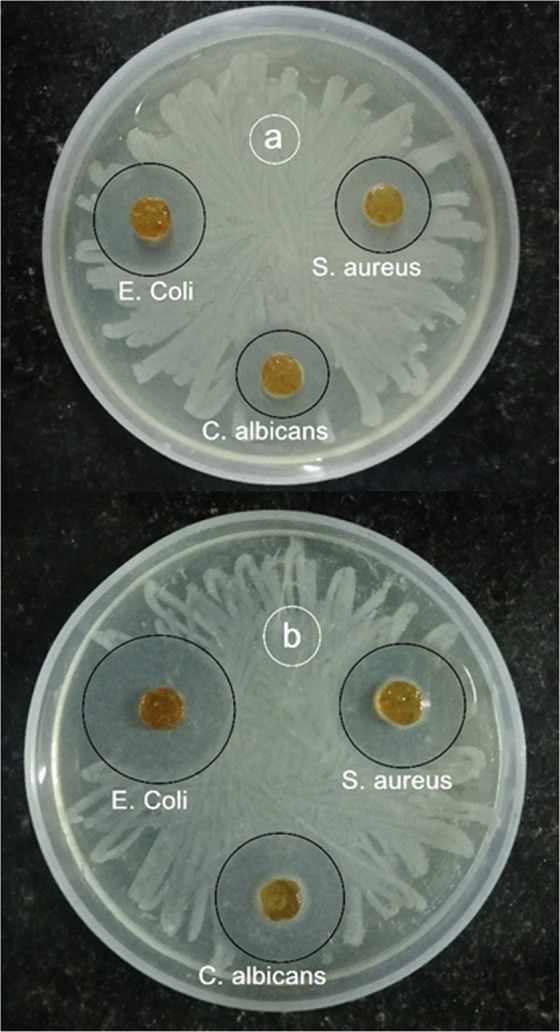
Microbial zone of inhibition (**a**) CS and (**b**) CS/1.5HANr composite.

The antimicrobial activities are the critical parameters to avoid bacterial infections and biofilm formation. The antibiogram of hydrogels are as shown (Fig. 9). Composite hydrogel showed superior inhibition against *Escherichia coli* and *Staphylococcus aureus* bacteria compared to the pristine CS hydrogel. The HANr reinforcement was more effective for *Escherichia coli* since 28% more inhibition was found for CS/1.5HANr compared to *Staphylococcus aureus*. One reason for this weak antibacterial activity against *Staphylococcus aureus* is due to the presence of outer multi-layered membrane, which consists of lipid and polysaccharide composed of O-antigen, outer and inner core formed by covalent bonds that is usually impermeable and blocks antibiotics^51^. However, *Escherichia coli* has a few layered cell walls composed of murein, teichoic acids and wall-associated surface proteins, which dissolves quickly in the presence of hydroxyl group of hydroxyapatite. Besides, few studies have reported that HANr cause increased Reactive Oxygen Species (ROS) production and activation of the inflammasome causes DNA damage, cell cycle delays, and apoptosis in mesothelial cells^52^. The antifungal activity of composite hydrogel is distinct; the interaction between phosphate ions (PO_4_^3−^) of hydroxyapatite can dissolve the outer membranes by oxidizing phospholipids of outer cell membrane of *Candida albicans* cause the changes the cellular morphology and cytoplasmic leakage leading to the death of pathogen^53,54^. Further, disc diffusion results support the absorbance values. Figure 10 shows the agar plates with a zone of inhibition (ZOI) against tested microbes. The ZOI concedes the susceptibility of the microbe towards the antibiotic. The width of the inhibition zone around the samples are summarized in Table 3 and all the samples had a zone of inhibition greater than 3 mm demonstrates antimicrobial activity was good level according to the standard SNV 195920-1992^34^.

**Table 3:** Inhibition area of microbial growth calculated around the samples through agar diffusion tests

pathogens	annular radius (mm)	
	CS	CS/1.5HANr
*Escherichia coli*	7.9 ± 0.2	9.8 ± 0.1
*Staphylococcus aureus*	6.6 ± 0.1	7.6 ± 0.2
*Candida albicans*	6.1 ± 0.2	8.1 ± 0.2

### Cytotoxicity assay

**Figure 11:**
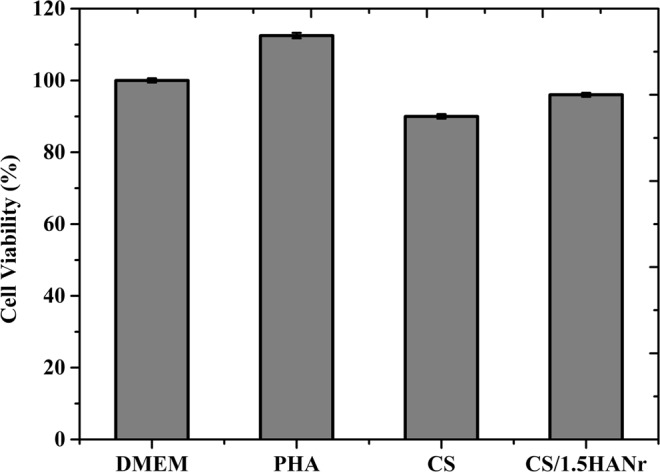
L929 mammalian cell viability on CS/1.5HANr composite hydrogel towards DMEM, PHA, CS and CS/1.5HANr composite samples after 72 h culture.

**Figure 12:**
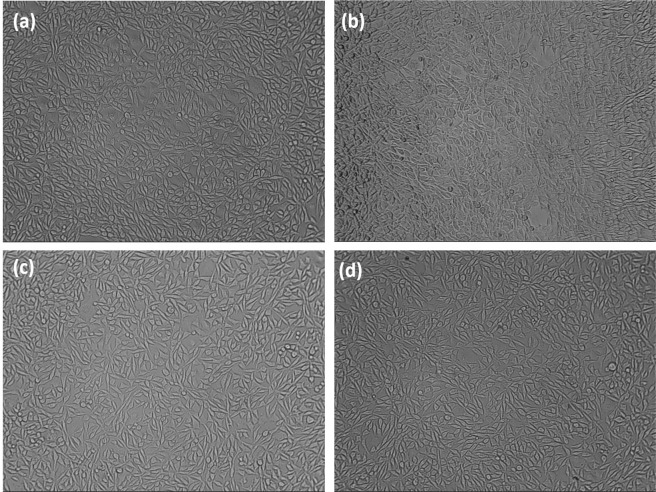
The morphological observations of the inverted microscopic images of L929 cells cultured for 72 h culture on (**a**) DMEM (**b**) PHA (**c**) CS and (**d**) CS/1.5HANr.

The *in vitro* cytotoxicity of the composite hydrogel towards L929 cell lines was tested using MTT assay. The mammalian cell viability of CS, CS/1.5HANr, DMEM and Phytohaemagglutinin (PHA) treated samples are as shown (Fig. 11). The potential cytotoxicity of CS and CS/1.5HANr hydrogels was estimated and compared with the control. PHA exhibits the highest cell viability (112%) and CS/1.5 HANr (96 ± 0.41%) show better cell proliferation than pristine CS (90 ± 0.63%). It could be due to the release of Ca^2+^ and PO_4_^3−^ bioactive ions from hydroxyapatite which anchors chitosan hydrogel might afford a tissue-like environment for cell adhesion and proliferation^55,56^. As per ISO standards for biocompatibility evaluation, composite hydrogel did not show any cytotoxicity to L929 mouse fibroblast cells, since its relative viability was more than 70%^57^. Further, the inverted microscopic images provide a preliminary understanding of the cellular response to hydrogels (Fig. 12). It was found that L929 mouse fibroblast cells exhibited a fusiform morphology and most of the cells spread and proliferate well on the culture plate. These results indicated that developed hydrogels showed no apparent cytotoxicity to L929 within incubation for 72 h and are the potential biomaterials with excellent antibacterial activities without significant cytotoxicity towards mammalian cells.

## Conclusions

In the present study, hydroxyapatite nanorod reinforced chitosan composite hydrogels were developed successfully with potential application as articular cartilage regeneration. Developed hydroxyapatite has a one-dimensional rod-like structure with an average of 186 ± 12  nm diameter and 1000–1500 nm length. The porous structure with interconnected porosity in the composite hydrogel was observed both on the surface and in cross-section. It could be essential for the exchange of nutrients and metabolic wastes as well fluid load support in diarthrodial joints. The swelling strength could decrease as a function of hydroxyapatite indicating that reinforcement influences the additional crosslinking and it was verified by FTIR spectra. The wettability values of hydrogels are optimum which supports the fibroblast growth. An essential improvement in the mechanical properties was found when HANr was incorporated into the CS matrix and the best results were found in CS/1.5HANr composite gels. Similar observations were found for viscoelastic properties which are relevant for the proposed application. The microbial retentively of composite hydrogel against tested microbes was satisfactory and incorporation of HANr to hydrogel increases the biocompatibility showing more than 90% cell viability responses of L929 fibroblasts after 72 h of culture. Agreeing with the above results and discussion, CS/1.5HANr composite hydrogel is the potential biomaterial for tissue engineering cartilage regeneration.

## Supplementary information

SUPPLEMENTARY INFO
