# Supplementary material for: Nanohydroxyapatite Reinforced Chitosan Composite Hydrogel with Tunable Mechanical and Biological Properties for Cartilage Regeneration
Source: Sci Rep. 2019 Nov 4;9:15957. doi: 10.1038/s41598-019-52042-7 (PMC6828803; doi:10.1038/s41598-019-52042-7)

Nanohydroxyapatite Reinforced Chitosan Composite Hydrogel with Tunable Mechanical and Biological Properties for Cartilage Regeneration

B.Y. Santosh Kumar^1^, Arun M. Isloor^2*^, G. C. Mohan Kumar^1*^, Inamuddin^3,4,5*^, Abdullah M. Asiri^3,4^

^1^Polymer Composites Laboratory, Department of Mechanical Engineering, National Institute of Technology Karnataka, Surathkal, Mangalore-575 025, India

^2^Membrane Technology Laboratory, Department of Chemistry, National Institute of Technology Karnataka, Surathkal, Mangalore-575 025, India

^3^Chemistry Department, Faculty of Science, King Abdulaziz University, Jeddah 21589, Saudi Arabia

^4^Centre of Excellence for Advanced Materials Research, King Abdulaziz University, Jeddah 21589, Saudi Arabia

^5^Advanced Functional Materials Laboratory, Department of Applied Chemistry, Faculty of Engineering and Technology, Aligarh Muslim University, Aligarh- 202 002, India

*Corresponding authors: Arun M. Isloor (isloor@yahoo.com) Inamuddin ([inamuddin@rediffmail.com](mailto:inamuddin@rediffmail.com)), G.C. Mohan Kumar ([mkumargc@gmail.com](mailto:mkumargc@gmail.com))

**Hydroxyapatite JCPDS: 00-009-0432**

**Name and formula**

Reference code: 00-009-0432

Mineral name: Hydroxylapatite, syn

PDF index name: Calcium Phosphate Hydroxide

Empirical formula: Ca5HO13P3

Chemical formula: Ca5 ( PO4 )3 ( OH )

**Crystallographic parameters**

Crystal system: Hexagonal

Space group: P63/m

Space group number: 176

a (Å): 9.4180

b (Å): 9.4180

c (Å): 6.8840

Alpha (°): 90.0000

Beta (°): 90.0000

Gamma (°): 120.0000

Calculated density (g/cm^3): 3.16

Measured density (g/cm^3): 3.08

Volume of cell (10^6 pm^3): 528.80

Z: 2.00

RIR: -

**Subfiles and Quality**

Subfiles: Inorganic

Mineral

Common Phase

Quality: Indexed (I)

**Comments**

Color: Green, bluish green, yellow-green, grayish green, violet, violet-blue, violet, colorless, light greenish white, gray, brown, pinkish red, pinkish-red, blue

General comments: I/I1 are peak values from a pattern which shows slight broadening of prism reflections.

Validated by calculated data 24-33.

Sample source: Sample obtained following the procedure indicated by Hodge et al., *Ind. Eng. Chem. Anal. Ed.*, **10** 156 (1938).

Optical data: B=1.651, Q=1.644, Sign=-

Additional pattern: To replace 34-10.

**References**

Primary reference: de Wolff, P., Technisch Physische Dienst, Delft, The Netherlands.

Optical data: **II**, 879

**Peak list**

No. h k l d [A] 2Theta[deg] I [%]

1 1 0 0 8.17000 10.820 12.0

2 1 0 1 5.26000 16.842 6.0

3 1 1 0 4.72000 18.785 4.0

4 2 0 0 4.07000 21.820 10.0

5 1 1 1 3.88000 22.902 10.0

6 2 0 1 3.51000 25.354 2.0

7 0 0 2 3.44000 25.879 40.0

8 1 0 2 3.17000 28.127 12.0

9 2 1 0 3.08000 28.967 18.0

10 2 1 1 2.81400 31.774 100.0

11 1 1 2 2.77800 32.197 60.0

12 3 0 0 2.72000 32.902 60.0

13 2 0 2 2.63100 34.049 25.0

14 3 0 1 2.52800 35.481 6.0

15 2 1 2 2.29600 39.205 8.0

16 3 1 0 2.26200 39.819 20.0

17 2 2 1 2.22800 40.453 2.0

18 3 1 1 2.14800 42.030 10.0

19 3 0 2 2.13400 42.319 4.0

20 1 1 3 2.06500 43.805 8.0

21 4 0 0 2.04000 44.370 2.0

22 2 0 3 2.00000 45.306 6.0

23 2 2 2 1.94300 46.713 30.0

24 3 1 2 1.89000 48.104 16.0

25 3 2 0 1.87100 48.624 6.0

26 2 1 3 1.84100 49.469 40.0

27 3 2 1 1.80600 50.494 20.0

28 4 1 0 1.78000 51.285 12.0

29 4 0 2 1.75400 52.102 16.0

30 0 0 4 1.72200 53.145 20.0

31 1 0 4 1.68400 54.442 4.0

32 3 2 2 1.64400 55.881 10.0

33 3 1 3 1.61100 57.129 8.0

34 5 0 1 1.58700 58.075 4.0

35 4 2 0 1.54200 59.940 6.0

36 3 3 1 1.53000 60.459 6.0

37 2 1 4 1.50300 61.662 10.0

38 5 0 2 1.47400 63.013 12.0

39 5 1 0 1.46500 63.445 4.0

40 3 0 4 1.45200 64.080 13.0

41 5 1 1 1.43300 65.033 9.0

42 4 2 2 1.40700 66.388 4.0

43 5 1 2 1.34800 69.701 3.0

44 4 3 1 1.31600 71.653 5.0

45 5 2 0 1.30600 72.288 4.0

46 4 2 3 1.28000 73.997 7.0

47 3 2 4 1.26500 75.025 3.0

48 2 1 5 1.25700 75.586 9.0

49 4 3 2 1.24900 76.156 1.0

50 5 1 3 1.23500 77.177 11.0

51 5 2 2 1.22100 78.230 9.0

**Stick Pattern**


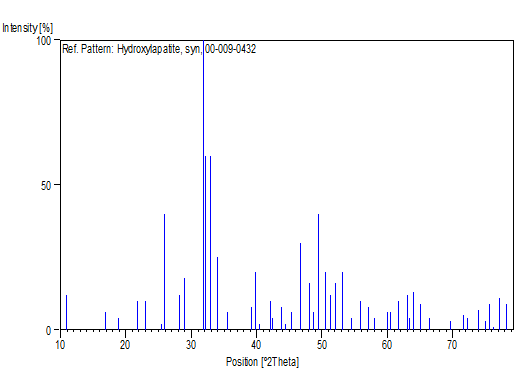


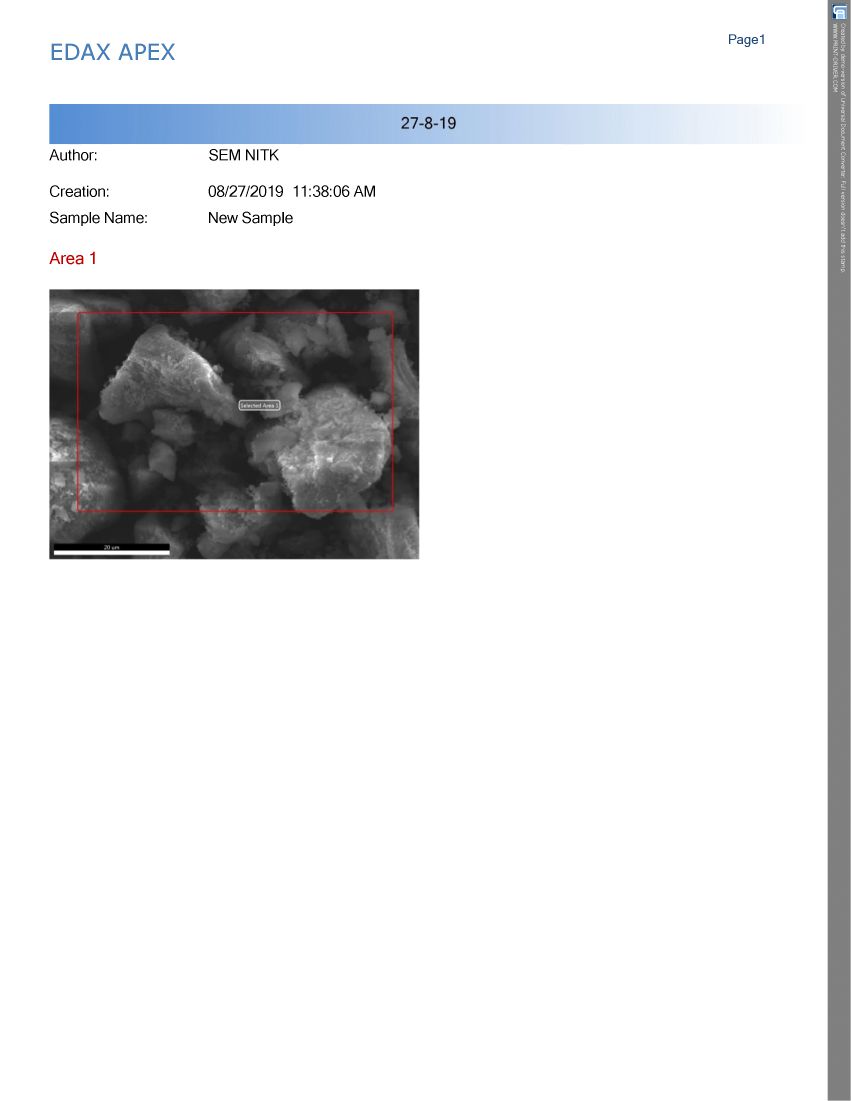

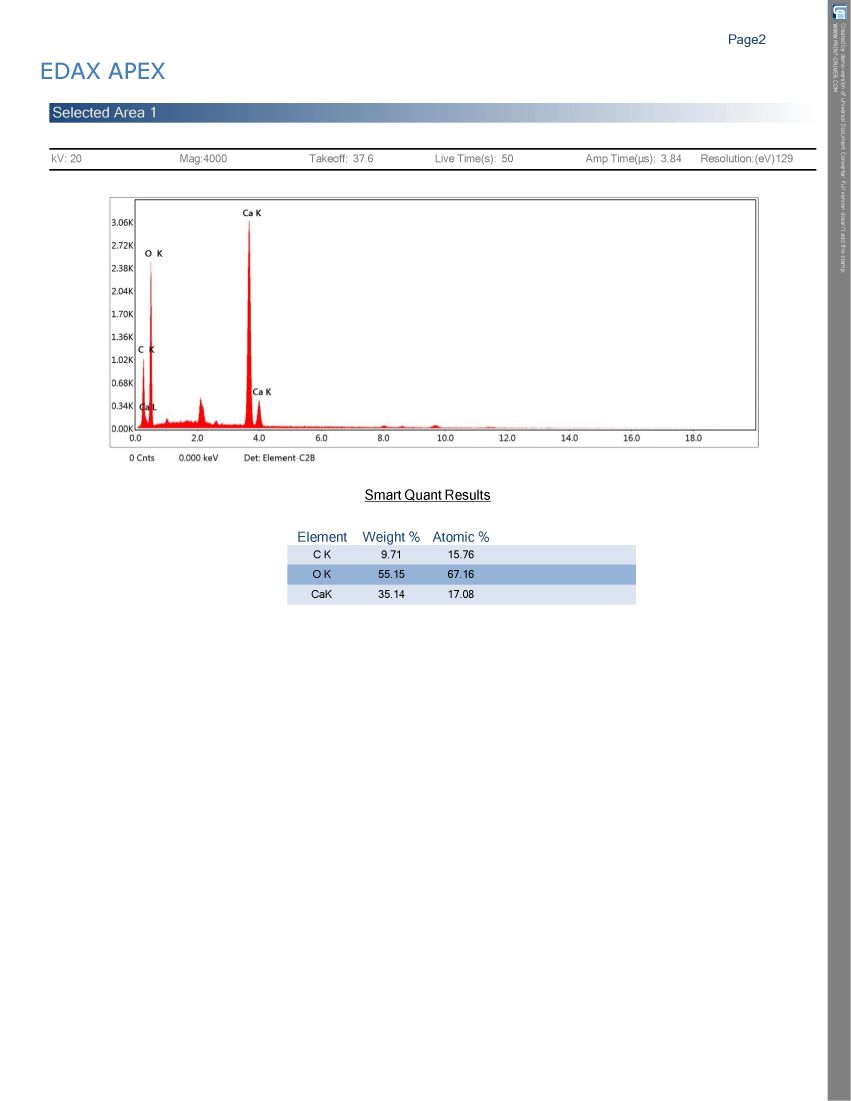

Supplement: Supplementary file 1 — SUPPLEMENTARY INFO [file 41598_2019_52042_MOESM1_ESM.docx]
